# Asthma and Other Respiratory Diseases of Children in Relation to Personal Behavior, Household, Parental and Environmental Factors in West China

**DOI:** 10.3390/toxics11120964

**Published:** 2023-11-28

**Authors:** Changan Cao, Yuna Wang, Li Peng, Weiqi Wu, Huimin Yang, Zhigang Li

**Affiliations:** 1 School of Medicine, Xiamen University, Xiamen 361104, China; changan_cao@163.com; 2 School of Chemical and Environmental Sciences, YiLi Normal University, Yining 835000, China; yunawang99@163.com (Y.W.);; 3 School of Energy and Environmental Engineering, University of Science of Technology Beijing, Beijing 10083, China; 42026184@xs.ustb.edu.cn; 4 Department of Geography, University College London, London WC1E 6BT, UK; zczqwwu@ucl.ac.uk; 5 Chinese Research Academy of Environmental Sciences, Beijing 100012, China

**Keywords:** respiratory disease, children, breast feeding, doing physical exercise, heating duration, opening window

## Abstract

Asthma and other respiratory diseases, which are of great concern in public health, are paid less attention in areas that are less economically developed. This research aimed to study the prevalence of critical respiratory diseases of children living in West China and figure out the potential influencing factors. A total of 575 children under the age of 14 were recruited from Xinjiang, China, to participate in the study in 2022. Information on activity patterns, socioeconomic and parental factors, and household and surrounding environment situations was obtained using a questionnaire survey. Logistic regression models were applied to estimate the odds ratios of respiratory disease prevalence in relation to behavior patterns, household, parental and environmental factors, respectively. The prevalence of ever doctor-diagnosed asthma, doctor-diagnosed bronchitis and current bronchitis were 4.7%, 19.0% and 14.4%, respectively. The prevalence of doctor-diagnosed pneumonia was 8.2%, which was two times higher in urban than rural areas. Longer annual heating duration was significantly associated with higher risks in children’s asthma and bronchitis, with an odds ratio (OR) and 95% confidence interval (95% CI) of 3.363 (95% CI: 1.215–9.298) and 1.267 (95% CI: 1.002–1.601), respectively. Opening the window longer in autumn would lead to higher risks of bronchitis, with ORs of 1.165 and 1.133, respectively, for doctor-diagnosed bronchitis and current bronchitis. Residential air pollution and having a residence close to waste incineration plant or garbage station were, respectively, significantly associated with higher risks of doctor-diagnosed bronchitis and asthma. Parental disease history was associated with a higher prevalence of children’s asthma and respiratory diseases, whereas breastfeeding and doing physical exercise were, respectively, significantly associated with a lower risk of asthma. A high prevalence of respiratory diseases in children in West China may be partly attributed to longer annual heating time, opening windows longer in autumn, surrounding environmental pollution, as well as parental disease history, whereas promoting physical activity and breastfeeding could be an effective measure to reduce the risk of childhood asthma in West China.

## 1. Introduction

In both developed and developing countries, respiratory diseases such as asthma have been confirmed as risk factors for further epidemics such as cardiovascular disease and cancer [1,2]. In 2019, chronic respiratory diseases were the third leading causes of death, responsible for 4.0 million deaths with a prevalence of 454.6 million cases globally [3]. A study found that there were 2.26 million new cases of trachea, bronchus and lung cancer in 2019 and the incidence was continuing to rise [4]. Children have a higher susceptibility to the adverse health effects of external factors due to their immature immune systems, their underdeveloped lung and metabolic systems, as well as high ventilation rates and mouth breathing, leading to an increased risk of impaired lung function and respiratory diseases [5]. A 10-year retrospective study started in 2009 based on one of the largest children’s teaching hospitals in China reported that bronchitis with a prevalence of 27.6%, pneumonia (18.5%), pneumonia affecting other systems (18.4%), and asthma and status asthmaticus (10.7%) were the top four most prevalent respiratory diseases and accounted for 75.2% of all recorded respiratory disease diagnoses [6]. It is reported that the prevalence of asthma in children increased from 0.91% in 1990 to 2.12% in 2010 [7]. However, a study conducted in Lanzhou, China, has reported that the prevalence of asthma decreased from 3.2% in 1994–1995 to 1.5% in 2018 [8]; a contemporary study conducted in Wuhan, China, has indicated that the prevalence of asthma was 2.5% and 2.4% and that of bronchitis was 27.1% and 29.8%, in 1994–1995 and 2018, respectively [9]. It seems currently difficult to assess accurate trends in the prevalence of respiratory diseases among children in China, due to a lack of comprehensive investigations or follow-up studies and insufficient evidence, especially in economically underdeveloped areas. Therefore, it is of great significance to investigate the prevalence of respiratory diseases and their risk factors among children, in order to take timely health risk prevention measures accordingly.

The etiology of respiratory diseases is quite complex and involves interactions between genetic factors, indoor and outdoor environmental exposures, and sociodemographic characteristics. Some studies have shown that indoor air pollution is of greater concern than outdoor air pollution, which is the third largest burden of diseases globally [10,11]. The combustion of solid fuels (biomass and coal) for heating and cooking is the main source of indoor air pollution in central countries [12]. Ambient air pollution [13], household molds [8] and indoor solid fuel burning [9] are evidenced as having negative impacts on asthma outcomes. Existing studies documented that the household dampness and mold occurred in 1/4 of children’s houses in southern China, and the presence of dampness and mold at home was associated with an increased risk of developing asthma (OR = 1.31, 95% CI: 1.08–1.59) in children [14,15]. A study conducted in Lanzhou, China, has illustrated that paternal asthma (OR = 3.143, *p* < 0.01) and maternal asthma (OR = 3.542, *p* < 0.01) were both associated with an increased prevalence of childhood asthma [8]. It is worth mentioning that several factors which have positive effects or increasing positive effects on the development of respiratory diseases are also uncovered in many studies. A study focused on the changes in asthma prevalence and risk factors has indicated that breastfeeding has changed to become a significant protective factor from 1994–1995 to 2018 [8]. A study has demonstrated that physical activity could reduce the risk of developing asthma [16], and a study based on meta-analysis has indicated that asthmatic children are less physically active than their healthy peers [17]; however, there is another study which showed no difference [18]. Due to the variety in research design and methodology, the risk factors and the relationships between factors and outcomes may not be consistent in different studies, such as the relationship between doing physical activity and the risk of asthma [16,18]. More epidemiology studies with various study designs need to be carried out to reveal respiratory disease status, identify the potential detrimental factors, and obtain more definitive evidence.

To date, the studies on respiratory diseases and their influencing factors are mostly focused on western countries, as well as mainland China in areas with developed economies or large population [9,10,11,12,13,14,15]; only a few studies have been conducted in areas that are economically underdeveloped or have a low population density [8]. This situation is neither conducive to rationally analyze the incidence of respiratory diseases nor beneficial to provide scientific evidence for reducing the risk of respiratory diseases. The Xinjiang Uygur Autonomous Region is regarded as the third lowest population density in China and the largest area located in West China. It has experienced rapid economic development and population increase in recent years, accompanied with some environmental pollution, under the implementation of The Western Development Strategy and The Belt and Road Initiative issued by the central government [19]. However, the prevalence of respiratory diseases of children in Xinjiang, as well as their predominant risk factors still remain unknown, in recent years with the rapid growth of economy and population. Thus, a cross-sectional study focusing on the prevalence of asthma and other respiratory diseases and the potential risk factors on children from Xinjiang, China, was conducted in 2022. This study paid attention to asthma, pneumonia and bronchitis, and concentrated on the potential influencing factors including behavior patterns, household, parental and environmental factors. The study was expected to add information for a more comprehensive understanding of the state and influencing factors of respiratory diseases in children in China.

## 2. Methods

### 2.1. Study Site and Participants

The survey was conducted in the whole region of Xinjiang Uygur Autonomous Region of China. Xinjiang has a typical temperate continental arid climate with sparse precipitation and large evaporation, with an average annual precipitation of 177.3 mm [20]. As a key area of the Western Development Strategy of China, Xinjiang has undergone substantial changes in population, socioeconomic and living environment due to rapid urbanization and industrialization over the past 20 years.

During the survey, an introduction regarding the objective of the study and the main contents in the questionnaire, a blank informed consent which informed participants of the voluntary and noncompulsory nature of the participation for each subject, as well as a blank questionnaire, were bundled together and were randomly handed out in different schools and communities. Each valid questionnaire received required a corresponding signed informed consent form. Finally, 575 pairs of valid signed consent forms and fulfilled questionnaires were obtained. Thus, 575 locally born children aged from 0 to 14 years old were enrolled in the study. The study protocol was reviewed and approved by the Scientific Research Ethics Committee, Chinese Research Academy of Environmental Sciences (Project reference No: 008-2021).

### 2.2. Questionnaire Survey and Data Collection

The questionnaire used in the study was designed by the American Thoracic Society Epidemiologic Standardization Project [21], which has been proved to be valid in previous research studies [8,22]. Additionally, on the basis of an investigation of newly released publications, some factors which would be potential emerging risk factors to respiratory diseases were added in the questionnaire. The questionnaire consisted of six parts: (1) the basic information of children, mainly including the child’s name, age, sex, weight and residence; (2) children’s household information, which mainly included the building types, cooking fuels and heating fuels at home, the ventilation modes of the kitchen, whether there were pets with fur, whether the house was decorated recently, whether mosquito coil, air purifiers and air fresheners were used, whether there was mold at home, the duration of heating, the duration of opening windows and using air conditioning in different seasons, etc.; (3) information about children’s physiology and health, including whether the child was premature and breastfed, whether the child had been diagnosed with respiratory diseases recently and in the past, etc.; (4) information of the child’s parents, including the parents’ education levels and occupations, smoking history and respiratory diseases history of the parents, etc.; (5) a child’s personal behavior patterns, e.g., whether they do physical exercise, whether they are exposed to environmental tobacco smoking (ETS), whether they are exposed to air pollution in commuting; (6) the residence’s surrounding environment conditions, such as whether the house was near the main road or close to waste incineration plants and waste recycling stations, residential air quality, and environmental dry conditions in different seasons.

### 2.3. Definition of Respiratory Diseases and Variables

Children’s respiratory diseases, including current bronchitis, ever doctor-diagnosed asthma, ever doctor-diagnosed bronchitis, and ever doctor-diagnosed pneumonia, were determined based on various questions. Taking ever doctor-diagnosed asthma as an example, ever doctor-diagnosed asthma was obtained by the question of ‘Has a doctor ever diagnosed asthma in your child?’ The participants were classified as having asthma when they answered “yes”.

The types of parental occupations included white collar (e.g., governmental office workers, doctors), blue collar (e.g., factory workers, and maintainers) and other occupations without regular income. Parental education level was classified by below college level and with or above college level. Whether a child did physical exercise was evaluated by the questions of “Do you do physical exercise in school?” and “Do you do physical exercise out of school?” Environmental tobacco smoking exposure of the child was defined if any member living with the child smoked currently. Air pollution exposure in commuting was assessed by the answers of on foot, bicycle and motorbike to the question of “What is the main way of transportation to and from school?” Household coal fuel use was determined by the answers of coal, and coal mixed with other fuels to the questions of “What is the main fuel type for cooking?” and “What is the main fuel type for heating?” Kitchen style was classified by separated kitchen and enclosed kitchen (attached to the living room). Whether there was air pollution in residential area was determined by the question of “How about the air quality of the children’s living area?” The answers of “slight pollution”, “moderate pollution” and “heavy pollution” were combined to define that there was air pollution in the living area. The distance of the residence to the main road was set as a binary variable: 0–0.5 km and above 0.5 km. Whether the residence was close to a waste incineration plant or garbage station was obtained from the question of “Whether your family is close to an incineration plant, garbage station, sewage ditch?” 

### 2.4. Statistical Analysis

Data were tested for normality (quantile–quantile plot) and homogeneity (Bartlett’s test for unequal variances). Continuous variables (e.g., weight) were present as the mean ± the standard deviation (SD), and categorical variables (e.g., district) were presented as the number (percentage) in each subgroup. Wilcox’s test and Pearson’s chi-square (χ^2^) test were applied to analyze the difference between continuous variables and between categorical variables, respectively. The associations of each factor with children’s respiratory diseases were assessed by binary logistic regression models and were shown with adjusted odds ratios (ORs) with 95% confidence intervals (95% CI), after controlling confounding factors. The statistical significance was defined when a *p* value was <0.05. All analyses were performed using SPSS software for Windows, version 23 (SPSS Inc., Chicago, IL, USA).

## 3. Results

### 3.1. Characteristics of Anthropometric, Personal Behavior Patterns and Parental Factors

The characteristics of the participants stratified by various factors are shown in Table 1. The average age of the children was 9.5 years old, and most of the children were 7–14 years old, accounting for 79.8% of the total participants. The children were roughly evenly distributed by gender and region. A larger portion of children were breastfed in infancy (93.6%). The rate of preterm birth among children was 5.9%. Among the participants, 74.6% of children did physical exercise more than 30 min/day. A total of 68.9% of children were found to have ETS exposure, while 67.0% were exposed to air pollution during commuting. Most of the children had their own beds or own rooms (63.5% and 75.1%, respectively), and lived in an apartment (75.5%). 

The proportions of children’s mothers and fathers with higher education level (with or above college) were both near 70%. A high proportion of white-collar workers were both found among children’s mothers and fathers (both near 70%). Compared with maternal smoking, the incidence of paternal smoking was very high. The prevalence of asthma in fathers and mothers were both about 2%, and 7–8% of children’s parents had a history of bronchitis. 

### 3.2. Characteristics of Household and Environmental Factors

As shown in Table 1, coal was used in 13.4% of households for cooking or heating. Most families used ventilation devices during cooking (92.0%). Of all the households, 23.3% were renovated in one year and 31.0% had the presence of mold. The portions of households using air fresheners and air purifiers were 52.5% and 46.6%, respectively. More than 60% of households had pets and used mosquito repellent incense. The average heating time was 4.8 months per year, and the average time of opening windows in the summer was 9.4 h per day, followed by the times in spring and autumn which were both 6.7 h per day. Similarly, the time of using air conditioning was highest in the summer (8.0 h per day), followed by the winter. About one quarter of children lived in areas with air pollution. A total of 57.4% of the households were within 500 m of main roads, and 8.7% were close to a waste incineration plant or garbage station or sewage ditch. 

### 3.3. Prevalence of Asthma and Other Respiratory Diseases

Table 2 shows the prevalence of ever “doctor-diagnosed” asthma, ever “doctor-diagnosed” and current (last 12 months) reported bronchitis, and ever “doctor-diagnosed” pneumonia. The prevalence of doctor-diagnosed asthma was 4.7%, which was 3.0% and 6.5% in urban and rural areas, respectively. The prevalence rates of doctor-diagnosed and current bronchitis were about 19% and 14.4%, respectively, which were comparable in rural and urban areas. The prevalence of doctor-diagnosed pneumonia was 8.2% and had approximately halved from urban to rural areas (10.7% vs. 5.4%). As shown in Table 2, the risk of ever doctor-diagnosed pneumonia in children was significantly higher in urban than rural areas, after adjusting confounding factors.

### 3.4. Effects of Behavior Patterns, Parental, Household and Environmental Factors on the Risks of Asthma and Other Respiratory Diseases 

Table 3 illustrates the ORs of asthma, bronchitis and pneumonia of children in association with personal behavior patterns, household, parental and environmental factors. The results show that boys were ever less likely to develop bronchitis than girls, with an OR of 0.472 (95% CI: 0.245–0.907). The children who lived in urban areas were significantly more likely to develop pneumonia (OR = 3.008, 95% CI: 1.149–7.873). The breastfed children were less likely to develop asthma, especially for the children who had been breastfed for 4–6 months (OR = 0.002, *p* < 0.05). No associations regarding the age, ETS exposure, preterm birth, body weight and air pollution exposure during commuting were observed for asthma and other respiratory diseases. However, a protective effect of doing physical exercise was observed on asthma among the children who did physical exercise no less than 30 min per day. 

Children whose mother had ever had asthma were more likely to have asthma (OR = 59.446, *p* < 0.05) and current bronchitis (10.886, *p* < 0.05), and children whose mothers had ever experienced bronchitis had significantly higher ORs in doctor-diagnosed pneumonia, doctor-diagnosed bronchitis and current bronchitis (*p* < 0.01 for all diseases). Furthermore, for the children whose fathers had ever gotten bronchitis, the ORs of doctor-diagnosed bronchitis and current bronchitis were 16.298 (*p* < 0.01) and 5.762 (*p* < 0.01), respectively. 

Children living in homes with a longer heating time per year had higher risks of developing asthma and bronchitis, with ORs of 3.362 (95% CI: 1.215–9.298) and 1.267 (95% CI: 1.002–1.601) for doctor-diagnosed asthma and bronchitis, respectively. Household coal use and household mold were potential risk factors for pneumonia, since the ORs of 0.152 (95% CI: 0.042–0.550) and 8.834 (95% CI: 2.418–32.572) were found for doctor-diagnosed pneumonia in the children whose family did not use coal for heating or cooking and the children whose houses had mold presence for more than 3 months, respectively. Additionally, using air conditioning for a longer time in the spring had a positive effect on the development of asthma, since the OR of doctor-diagnosed asthma was 0.253 (95% CI: 0.074–0.867). Opening windows for a longer time in the autumn could be a risk factor for bronchitis in children, with ORs of 1.165 (95% CI: 1.041–1.303) and 1.133 (95% CI: 1.005–1.276) for ever doctor-diagnosed bronchitis and current bronchitis, respectively. However, a longer window opening time in spring was related to a lower risk of respiratory diseases, although no statistical significance was found. As shown in Table 4, the interaction between window opening duration and annual heating time showed that opening windows for a longer time in spring could reduce the risks of doctor-diagnosed asthma and bronchitis. No associations were found between other factors such as household decoration, having pets, using mosquito-repellent incense and air fresheners, with asthma and other respiratory diseases (Table 3).

Furthermore, for the children who lived in the areas without air pollution, the OR of doctor-diagnosed bronchitis was 0.439 (95% CI: 0.223–0.864). It was observed that the long distance from home to the waste incineration plant, garbage station, odor ditch, etc., was a protective factor to the development of asthma in children. An OR of 0.001 (95% CI: 0.223–0.864) for doctor-diagnosed asthma was found in children who lived far away from the aforementioned places in a significant statistical significance. The distance from residence to main roads was not associated with the risk of respiratory diseases.

## 4. Discussion

In this study, we evaluated the prevalence of respiratory diseases such as asthma, bronchitis and pneumonia in children, in relation to personal activity patterns, parental, household and environmental factors in Xinjiang, the westernmost and the largest western region of China. A higher prevalence of ever doctor-diagnosed asthma in children was found in rural areas, but after controlling the potential confounding factors, the difference in the prevalence of ever doctor-diagnosed asthma between urban and rural areas showed no significance. However, there was a higher risk of doctor-diagnosed pneumonia in the children who lived in urban areas, in comparison with the children from rural areas after adjustment for potential confounders. The prevalence rates of doctor-diagnosed bronchitis and current bronchitis, were, respectively, comparable in rural and urban areas. Among various potential factors, breastfeeding, doing physical exercise no less than 30 min per day, using air conditioning longer in the spring, and having a mother with a higher education level showed positive effects on the respiratory diseases. In contrast, parental asthma and bronchitis histories, father highly educated, longer annual heating time, opening windows for a longer time in the autumn, household coal use, living in an urban area and in a moldy house, residing in areas with air pollution and having a residence close to the places with waste incineration plants and garbage stations and so on were observed to be potential risk factors for the higher prevalence of asthma and other respiratory diseases.

Many studies have attempted to evaluate the association between breastfeeding and the risk of respiratory diseases such as asthma in children [8,23,24,25,26]. Evidence from observational studies suggests that breastfeeding minimizes the pulmonary sequelae of respiratory infections, improves lung function in school-age children, and also protects lung function in children who are exposed to high levels of air pollution and passive smoking [23,25,26]. It suggests that breastfeeding appears to counteract the effects of environmental detrimental influences on lung development [27]. A birth cohort study of healthy infants found protective causal effects of exclusive breastfeeding on the risk of developing a lower respiratory tract infection in infancy and asthma and allergic rhinitis in childhood, which were mediated through interactions on the gut microbiota [28]. However, there is controversy in many studies in this field as to whether the duration of breastfeeding is associated with asthma [23,24,25,26,27,28]. In this study, we examined whether the child was breastfed and the duration of breastfeeding. Among the breastfed children, 31.6% and 33.1% of children were breastfed 4–6 months and more than 6 months, respectively, with an OR of 0.002 (*p* = 0.034) and 0.007 (*p* = 0.056) for doctor-diagnosed asthma. This finding was consistent with the results from several other studies [29,30,31,32,33,34]. Nutrients and bioactive factors in breast milk favor the formation of bodily immunity for optimal growth and development [32,33], especially during the first six months of an infant’s life. They also can have a contrasting effect on the development of immune function and subsequent susceptibility to allergic diseases, such as asthma [34,35]. There has been a study linking the protective effects of breastfeeding to reduced susceptibility to viral infections, revealing that infants fed formulas in comparison with breast milk had a higher incidence of atopic dermatitis and wheezing illnesses in early childhood and encouraging exclusive breastfeeding for at least 4 to 6 months [33]. It could be supported by the clinical practice guidelines internationally that exclusive breastfeeding for the first 4 to 6 months of life is recommended for the prevention of allergic disease in children such as asthma, particularly for the babies of atopic mothers [36]. Therefore, we concluded that children breastfed for 4–6 months would have a lower risk of developing physician-diagnosed asthma, although further insight into the breastfeeding duration–response relationship is required.

Physical activity is regarded as one of the most commonly reported symptom triggers in patients with asthma [37]. However, there is growing evidence that regular exercise and physical activity are associated with improved clinical and patient-reported outcomes [37,38,39]. A pilot study found that asthma patients tolerated and responded well to high-intensity interval training, and their aerobic adaptations were similar to those of their healthy peers [38]. A systematic review of the effect of physical activity on asthma outcomes indicates that physical activity can improve asthma control, quality of life, and inflammatory serologies [39]. In this study, we investigated the duration of regular physical exercise and found that doing physical exercise 30–60 min/day and more than 60 min/day were significantly associated with a lower risk of doctor-diagnosed asthma, with ORs of 0.015 (95% CI: 0.000–0.612) and 0.022 (95% CI: 0.001–0.722), respectively. Additionally, it showed an increasing positive effect of doing physical exercise with a longer time on the risk of asthma. Our finding was also supported by a systematic review which suggested that exercise appeared to favor improvements in aerobic fitness, asthma symptoms and quality of life [40]. It is generally assumed that exercise can induce dyspnea if in a natural state and without a proper control (such as using bronchodilators). However, regular exercise can bring about several benefits including a reduction in dyspnea, as well as the improvement of aerobic capacity and the number of exacerbations and the intensity of exercise-induced bronchoconstriction [41]. This is because exercise has a positive effect on inflammation, and acute exercise could cause the downregulation of glucocorticoid receptor expression in circulating leukocytes [42]. Respiratory diseases are largely attributed to inflammation, and asthma is the most common chronic inflammatory disease of the airway [43]. Consequently, current US guidelines for children and adolescents recommend as least 1 h of moderate-to-vigorous physical activity each day, with the majority of that time being spent on aerobic activity [44]. However, there has been a misconception that adolescents with asthma should be excluded from exercise in the past, which is evidenced by a study that indicated asthmatics typically had lower levels of physical activity compared to healthy patients, and that lack of physical activity was associated with increased morbidity and mortality [45]. Now, our study showed a lower risk of asthma with regular physical exercise, that was supported by increasing evidence [38,39].

Pneumonia, as one of the most serious and common infectious diseases in children, is a leading cause of pediatric mortality and morbidity worldwide [46]. China has experienced an extremely high prevalence of childhood pneumonia in urban areas over the past decades [47]. In this study, we also found a significant higher prevalence of pneumonia in urban rather than rural areas, with an OR of 3.008 (*p* = 0.025). This result was in line with a retrospective cohort study which was performed in Changsha, China, during 2019–2020 [48]. But the total prevalence of pneumonia (8.2%) in this study was lower than that (32.8%) of a cohort study [48]. It is accepted that pneumonia in children is related to various environmental factors [48,49]. A cross-sectional study conducted in eight cities of China has concluded that indoor environmental factors including mold and the use of solid fuels for cooking are risk factors for childhood pneumonia [49]. A significant association between household mold and childhood pneumonia was also found in our study (OR = 8.834, *p* = 0.042), which was evidenced in another study conducted in Urumqi, Xinjiang [50]. Many epidemiology studies have ascertained that pneumonia is a lower respiratory infection and could be caused by various infectious agents, such as viruses, bacteria and fungi [46,51]. Meanwhile, visible mold spots or moldy air are thought to be the result of excessive dampness and can promote the rapid growth of harmful microorganisms, including bacteria, fungi or virus [52]. Therefore, household moldy environment was revealed to have a strong dose–response relationship with childhood pneumonia [49]. 

Additionally, we found that household coal use for cooking or heating was significantly in association with childhood pneumonia, which was also consistent with other epidemiology studies [53,54]. Coal combustion emits lots of chemicals and particles, including nitrogen oxides, sulfur oxides, ozone, carbon monoxide, PM_2.5_, etc., which can irritate respiratory tracts and lungs, adversely affect the host’s defense systems against pathogens, and elevate the risk of respiratory tract infections [53]. Coal smoke may also increase the severity of respiratory infections by causing inflammation in pulmonary airways [55]. Strong evidence has been presented and indicated that respiratory health problems are associated with various pollutants from indoor solid fuel use [53,54,55]. A systematic review and meta-analysis in Africa, China and Latin America concluded that the risk of pneumonia in young children was increased by the exposure to unprocessed solid fuels combustion [54]. A retrospective cohort study in China, which provided evidence for the first time that indoor air pollution from solid fuel burning increased pneumonia deaths in adults, reported that coal usage was positively associated with pneumonia mortality [56], supporting the result in our study. To indicate the influences of household factors and residential regions on childhood pneumonia, we analyzed the prevalence of pneumonia in children classified by household coal use and mold. As shown in Table S1, among the children who ever got doctor-diagnosed pneumonia, higher rates of household coal use and presence of household mold were found in urban than rural areas, and we speculated this may be part of the reason why the prevalence of pneumonia was higher in urban than in rural areas.

We found that longer window opening time in spring, meaning longer ventilation, was in relation to a lower risk of respiratory diseases, although there’s no statistical significance. On the other hand, detrimental effects of opening windows for a longer time in autumn on the prevalence of doctor-diagnosed bronchitis and current bronchitis were observed. It was in line with a previous work conducted in Wuhan, China, which indicated that opening windows longer in the autumn was a risk factor for impaired lung function after adjusting for confounders [57]. However, using air conditioning for a longer time in spring showed a positive effect on the prevalence of asthma in this study. Considering the longer daylight hours and high temperature in autumn, and lower temperature in spring, we speculated that cool household condition in autumn and a warm indoor environment in spring would help to reduce the risk of bronchitis and asthma, respectively. In addition, longer annual heating time was observed as a significant risk factor for doctor-diagnosed asthma and bronchitis in this study. Since heating generally occurred in winter and spring, to better understand the interaction effects of annual heating time and ventilation time on the four respiratory diseases, the interactions of window opening time in winter, window opening time in spring and annual heating duration on the respiratory diseases were analyzed, respectively. As shown in Table 4, on the basis of the annual heating time, longer window opening time in spring would halve the risk of doctor-diagnosed asthma and lead to a reduced risk of doctor-diagnosed bronchitis. However, longer window opening time in winter was likely to significantly increase the risk of doctor-diagnosed pneumonia and may lead to high risks of doctor-diagnosed asthma and bronchitis, although no statistical significance was found. One plausible explanation was that longer window opening time in winter may decrease the household temperature and increase the indoor pollution via outdoor penetration [58]. Consequently, it posed higher risk to the respiratory health of children, since children are vulnerable to cold air and pollutants [10,58].

Similar to the study conducted in Wuhan, China, which indicated that maternal higher education level was associated with higher lung function level [57], we observed that the children whose mother had college or above education attainment had a lower risk of current bronchitis. But the opposite was found for father in this study. The potential explanation would be that a higher maternal education level, indicating higher socioeconomic condition, would mean good living standards and nutrition status, and good protection of children from diseases, while a higher paternal education level may mean a busy work schedule and less time and energy to take care of children. There may be a certain relationship between parental education level and childhood respiratory diseases, and further studies are needed to investigate the possible mechanisms underlying the association between these two. 

Asthma is a polygenic hereditary disease with an obvious tendency of family aggregation [59]. The epidemiology of childhood bronchial asthma family heritability shows that the heritability of first-degree relatives of asthmatic patients is about 80%, and the relative risks of first-degree relatives and sibling relatives are 7.39 and 4.47, respectively [60]. It can be seen that genetic factors are important reasons in the occurrence of asthma. In this study, we found maternal asthma and bronchitis were significantly associated with childhood asthma and bronchitis. This result was in accordance with that of a cross-sectional study in Lanzhou, China [8], and a large population-based epidemiology study in Northeast China [13]. Moreover, a modification of breastfeeding to the effect of maternal asthma and bronchitis histories on children’s respiratory diseases was found in our study (shown in Table S2). In comparison with the children without breastfeeding, a lower prevalence of childhood asthma was observed among the children who were breastfed in spite of whether their mother got asthma or bronchitis histories. Additionally, we also found that girls seemed to be more susceptible to doctor-diagnosed bronchitis than boys. It has been shown that sex-specific hormonal changes influence respiratory diseases such as asthma and bronchitis, and boys are more likely to develop diseases during childhood [61]. However, this pattern changes after puberty and females are more likely than males to develop the disease [61]. Our study included more children aged 7–14 years (accounted for 79.8% of total, Table 1), precisely the age of puberty, which may result in girls being more susceptible. 

Furthermore, we found that residential air pollution and living near to a waste incineration plant or garbage station were in association with higher risks of respiratory diseases, particularly for doctor-diagnosed asthma and bronchitis. As far as we know, pollutants such PM_2.5_, ozone (O_3_), nitrogen oxides (NOx), sulphur oxides (SOx) and so on, contained in ambient air would lead to detrimental health effects to humans [10]. Waste incinerators are widely documented as a source of air pollutants including acid gases, NOx, SOx, heavy metals, particulates and persistent organic pollutants (POPs) such as dioxins and furans. Although we did not measure specific ambient pollutants in residences and areas nearby waste incineration plants, we investigated the overall ambient air quality state in the residential area and surveyed the distance of the residence to a waste incineration plant or garbage station. The results indicated that a large number of children lived in the areas with air pollution and lived close to the waste incineration plant or garbage station. Furthermore, much evidence suggests that 13% of global incidence of asthma in children could be attributed to air pollution and implies that air pollution has a negative impact on asthma outcomes [10,62]. 

This study had several limitations. First, we recruited a limited number of children to participate in the survey, and thus the findings may be missing generalizability. Second, this was a cross-sectional study based on a questionnaire survey, which may contain recall bias in the questionnaire filling process. In addition, we did not obtain data on indoor and outdoor air pollutant concentrations, which had widely been shown to affect respiratory health in children. We, thus, cannot specify the intrinsic relationship between children’s respiratory diseases and certain factors such as household coal use and window opening time. Furthermore, although the adjustment for confounders such as ETS, household coal use, ventilation use, etc., may partially mitigate the confounding of indoor air pollutants, we cannot exclude residual confounding by these or other factors. Despite these limitations, this study provided a valuable opportunity to realize the prevalence of respiratory diseases and understand the influencing factors of respiratory diseases in children of western China.

## 5. Conclusions

As one of few studies conducted in western China, we provided more evidence to realize the situation of childhood respiratory diseases and the potential influencing factors. We identified that the prevalence of children’s asthma, bronchitis and pneumonia in West China could not be ignored, which could be mainly attributable to parental respiratory disease history, longer annual heating time, longer window opening time in autumn, living in air polluted areas and close to a waste incineration plant or garbage station. It reminded us not to ignore the respiratory health problems of children living in polluted areas and near waste incineration plants. Fortunately, we ascertained that breastfeeding and doing physical exercise had played a positive role in the reduction of respiratory disease risk, particularly in the risk of childhood asthma. Thus, appropriate duration of breastfeeding and physical exercise should be strengthened to reduce the risk of childhood respiratory diseases in western China.

## Figures and Tables

**Table 1:** Anthropometry, sociodemographic, household and parental information and children’s behavior patterns.

Category	Variables	Total (n = 575)	Variables	Total (n = 575)
Anthropometry	Age, years	9.5 ± 3.0	Male, n (%)	298 (51.8%)
	Age, years		Urban, n (%)	299 (52.0%)
	0–3	20 (3.5%)	Weight, kg	34.5 ± 14.0
	4–6	96 (16.7%)	Breast feeding, n (%)	538 (93.6%)
	7–14	459 (79.8%)	Preterm birth, n (%)	34 (5.9%)
Socioeconomic factor	Sleep in own room, n (%)	365 (63.5%)	Father education level, ≥College, n (%)	386 (68.4%)
	Sleep in own bed, n (%)	432 (75.1%)	Mother education level, ≥College, n (%)	383 (67.9%)
	Paternal occupation, n (%)		Maternal occupation, n (%)	
	White collar ^1^	380 (67.4%)	White collar ^1^	383 (67.9%)
	Blue collar ^2^	114 (20.2%)	Blue collar ^2^	79 (14.0%)
	Others	70 (12.4%)	Others	102 (18.1%)
Personal activity pattern	Physical exercise time, min/day		ETS exposure, n (%)	396 (68.9%)
	≤30	146 (25.4%)	Air pollution exposure in commuting, n (%)	380 (67.0%)
	30–60	242 (42.1%)		
	>60	187 (32.5%)		
Parental factors	Paternal smoking, n (%)	331 (57.6%)	Maternal smoking, n (%)	11 (2.0%)
	Paternal asthma, n (%)	10 (1.8%)	Maternal asthma, n (%)	11 (2.0%)
	Paternal bronchitis, n (%)	39 (6.9%)	Maternal bronchitis, n (%)	47 (8.3%)
Household factors	Building type, n (%)		Air fresheners use, n (%)	302 (52.5%)
	Bungalow	19.7%	Air purifier use, n (%)	268 (46.6%)
	Department	75.5%	Heating duration, months	4.8 ± 1.5
	Villa	4.9%	Window opening time in spring ^3^	6.7 ± 5.3
	Household coal use, n (%)	77 (13.4%)	Window opening time in summer ^3^	9.4 ± 6.8
	Ventilation use for cooking, n (%)	529 (92.0%)	Window opening time in autumn ^3^	6.7 ± 5.1
	Enclosed kitchen, n (%)	418 (27.3%)	Window opening time in winter ^3^	3.3 ± 3.7
	Home renovation, n (%)	137 (23.8%)	Air conditioning use time in spring ^3^	1.1 ± 2.5
	Presence of pets, n (%)	366 (63.7%)	Air conditioning use time in summer ^3^	8.0 ± 6.0
	Presence of mold, n (%)	178 (31.0%)	Air conditioning use time in autumn ^3^	1.4 ± 2.7
	Mosquito coil use, n (%)	412 (71.7%)	Air conditioning use time in winter ^3^	3.9 ± 5.4
Environmental factors	Polluted air in living area, n (%)	147 (25.6%)	Dry humidity in spring, n (%)	174 (30.3%)
	Close to waste incineration plant, n (%)	50 (8.7%)	Dry humidity in summer, n (%)	223 (38.8%)
	Distance to main road within 500 m, n (%)	330 (57.4%)	Dry humidity in autumn, n (%)	268 (46.6%)
			Dry humidity in winter, n (%)	387 (67.3%)

Values are mean (SD) or number (percentage %). ^1^ “White collar” includes teacher, businessperson, clerk, housewife (few cases), and other non-manual laborer occupations. ^2^ “Blue collar” refers to factory worker, construction worker, building cleaning worker, farmer, and other manual laborer occupations. ^3^ The time is counted in hours/day.

**Table 2:** Prevalence of asthma and respiratory disease related symptoms of children, and the odds ratios (OR) and 95% confidence interval (95% CI) of the prevalence of the symptoms of children in urban area versus rural area.

Disease	Prevalence in Total, n (%)	Prevalence, n (%)			Odds Ratios (95% CI) and *p* Value	
		Urban Area	Rural Area	*p*-Value	OR (95% CI) ^1^	*p*-Value
Doctor-diagnosed asthma	27 (4.7%)	9 (3.0%)	18 (6.5%)	0.050 *	0.277(0.038, 2.028)	0.277
Doctor-diagnosed bronchitis	109 (19%)	55 (18.4%)	54 (19.6%)	0.750	0.753(0.399, 1.422)	0.753
Doctor-diagnosed pneumonia	47 (8.2%)	32 (10.7%)	15 (5.4%)	0.023 *	3.008 (1.149, 7.873)	0.025 *
Current bronchitis	83 (14.4%)	43 (14.4%)	40 (14.5%)	1.000	0.798 (0.404, 1.577)	0.516

* *p* < 0.05. Bold numbers were indicated as statistically significant. ^1^ Adjusted for gender, age, height, premature birth, parental education level, parental occupation, father smoking, mother smoking, sleeping in own room, sleeping in own bed, household coal use, ventilation use when cooking, breast feeding, window opening and air conditioning use in different seasons, home renovation, presence of pets, presence of mold, mosquito coin use, air freshener use, air purifier use, kitchen type, parental bronchitis, intake of fruit, dairy, seafood and high-calorie food, doing physical exercise, ETS exposure and air exposure during commuting.

**Table 3:** Adjusted odds ratios (OR) ^1^ of asthma and respiratory disease-related symptoms of children in relation to socioeconomic, parental and household factors.

Variables	Doctor-Diagnosed Asthma	Doctor-Diagnosed Bronchitis	Doctor-Diagnosed Pneumonia	Current Bronchitis
Sex (ref: female)	0.075 (0.004, 1.311)	0.472 (0.245, 0.907) *	0.895 (0.374, 2.144)	0.557 (0.282, 1.101)
Age (year)	0.667 (0.384, 1.159)	1.102 (0.957, 1.270)	0.955 (0.791, 1.153)	1.020 (0.882, 1.179)
District (ref: rural)	0.277 (0.038, 2.028)	0.753 (0.399, 1.422)	3.008 (1.149, 7.873) *	0.798 (0.404, 1.577)
ETS exposure (ref: yes)	0.142 (0.005, 3.974)	0.730 (0.341, 1.561)	1.009 (0.361, 2.816)	0.764 (0.342, 1.709)
Preterm birth (ref: yes)	0.293 (0.007, 12.750)	1.050 (0.242, 4.550)	0.829 (0.129, 5.314)	0.525 (0.131, 2.098)
Body weight (kg)	1.083 (0.968, 1.211)	0.992 (0.965, 1.019)	1.023 (0.987, 1.060)	1.006 (0.980, 1.034)
Breast feeding (ref: no)				
<4 months	0.263 (0.009, 7.695)	3.262 (0.551, 19.303)	0.339 (0.063, 1.833)	2.712 (0.415, 17.721)
4–6 months	0.002 (0.000, 0.642) *	1.887 (0.311, 11.468)	0.412 (0.075, 2.254)	1.634 (0.247, 10.833)
>6 months	0.007 (0.000, 1.126)	1.820 (0.304, 10.915)	0.521 (0.097, 2.800)	2.560 (0.389, 16.841)
Sleep in own room (ref: yes)	0.025 (0.000, 1.469)	0.621 (0.229, 1.680)	0.500 (0.113, 2.222)	1.225 (0.445, 3.368)
Sleep in own bed (ref: yes)	23.906 (0.413, 138.469)	1.710 (0.573, 5.104)	2.565 (0.543, 12.121)	0.635 (0.201, 2.003)
Air exposure during commuting (ref: yes) ^2^	0.175 (0.010, 3.125)	1.293 (0.655, 2.552)	1.412 (0.537, 3.713)	0.697 (0.322, 1.508)
Doing physical exercise (ref: <30 min/day) ^2^				
30–60 min	0.015 (0.000, 0.612) *	1.135 (0.496, 2.598)	0.415 (0.126, 1.364)	0.513 (0.219, 1.202)
>60 min	0.022 (0.001, 0.722) *	0.994 (0.402, 2.460)	0.887 (0.286, 2.746)	0.625 (0.257, 1.519)
Mother occupation (ref: no stable income)				
Mother being blue collar	0.074 (0.001, 5.360)	0.650 (0.330, 2.677)	0.983 (0.111, 8.679)	1.568 (0.395, 6.231)
Mother being white collar	12.904 (0.318, 523.918)	0.940 (0.330, 2.677)	1.995 (0.400, 9.954)	0.971 (0.300, 3.144)
Paternal occupation (ref: no stable income)				
Father being blue collar	14.136 (0.060, 333.067)	2.506 (0.546, 11.498)	0.374 (0.063, 2.214)	0.749 (0.169, 3.322)
Father being white collar	0.088 (0.000, 30.175)	3.304 (0.730, 14.959)	0.422 (0.093, 1.917)	0.968 (0.226, 4.137)
Paternal education (ref: <College)	10.831 (0.273, 429.215)	0.593 (0.192, 1.830)	1.705 (0.360, 8.081)	4.040 (1.179, 13.847) *
Maternal education level (ref: <College)	0.065 (0.002, 1.762)	0.703 (0.237, 2.084)	0.909 (0.178, 4.636)	0.272 (0.086, 0.864) *
Maternal asthma (ref: no)	59.446 (1.992, 177.403) *	5.151 (0.776, 34.172)	3.796 (0.193, 74.588)	10.886 (1.645, 72.045) *
Maternal bronchitis (ref: no)	0.284 (0.001, 147.149)	14.289 (4.818, 42.377) **	8.875 (2.418, 32.572) **	12.166 (4.161, 35.574) **
Paternal asthma (ref: no)	25.398 (0.005, 121.496)	2.120 (0.253, 17.738)	2.326 (0.146, 36.925)	0.630 (0.045, 8.812)
Paternal bronchitis (ref: no)	2.396 (0.002, 262.240)	16.298 (4.925, 53.938) **	1.143 (0.236, 5.530)	5.762 (1.961, 16.928) **
Building type (ref: bungalow)				
Apartment	0.242 (0.014, 4.050)	0.752(0.303, 1.867)	4.397 (0.879, 21.983)	1.049 (0.384, 2.868)
Villa	1.041 (0.014, 77.985)	2.919 (0.639, 13.336)	5.423 (0.758, 38.813)	0.973 (0.172, 5.511)
Kitchen type (ref: enclosed)	0.592 (0.041, 8.604)	1.430 (0.715, 2.861)	0.987 (0.360, 2.706)	0.880 (0.410, 1.890)
Ventilation use when cooking (ref: no)				
Hood	10.018 (0.911, 110.228)	1.219 (0.316, 4.701)	0.594 (0.124, 2.847)	1.192 (0.264, 5.391)
Fan	0.730 (0.398, 37.172)	0.701 (0.116, 4.232)	0.000 (0.000, 0.000)	0.463 (0.054, 3.952)
Heating duration per year (month)	3.362 (1.215, 9.298) *	1.267 (1.002, 1.601) *	0.908 (0.652, 1.263)	1.201 (0.942, 1.531)
Household coal use (ref: yes)	0.797 (0.052, 12.136)	1.532 (0.549, 4.274)	0.152 (0.042, 0.550) **	1.303 (0.444, 3.822)
Window opening time in spring (hours/day)	0.701 (0.397, 1.237)	0.898 (0.793, 1.016)	0.987 (0.829, 1.175)	1.041 (0.930, 1.166)
Window opening time in summer (hours/day)	1.041 (0.832, 1.301)	0.968 (0.906, 1.033)	0.991 (0.899, 1.092)	0.961 (0.897, 1.031)
Window opening time in autumn (hours/day)	0.884 (0.607, 1.287)	1.165 (1.041, 1.303) **	0.937 (0.766, 1.147)	1.133 (1.005, 1.276) *
Window opening time in winter (hours/day)	2.703 (0.965, 7.570)	1.004 (0.851, 1.185)	1.119 (0.898, 1.396)	0.916 (0.776, 1.081)
Air conditioning use time in spring (hours/day)	0.253 (0.074, 0.867) *	0.844 (0.694, 1.026)	0.962 (0.698, 1.324)	0.915 (0.771, 1.086)
Air conditioning use time in summer (hours/day)	0.946 (0.762, 1.175)	0.972 (0.908, 1.041)	0.930 (0.833,1.037)	0.973 (0.911, 1.041)
Air conditioning use time in autumn (hours/day)	1.762 (0.980, 3.168)	1.102 (0.939, 1.295)	0.806 (0.568, 1.143)	1.150 (0.985, 1.344)
Air conditioning use time in winter (hours/day)	0.934 (0.639, 1.363)	1.002 (0.929, 1.080)	1.012 (0.905, 1.132)	0.965 (0.893, 1.042)
Decorate recently (ref: yes)	1.835 (0.086, 39.095)	0.978 (0.470, 2.035)	0.825 (0.291, 2.338)	0.690 (0.325, 1.464)
Have pets (ref: no)	2.899 (0.181, 46.517)	1.521 (0.761, 3.039)	1.245 (0.437, 3.549)	1.552 (0.739, 3.259)
Mold presence (ref: no)				
1 month	7.750 (0.316, 189.835)	1.344 (0.590, 3.059)	0.649 (0.178, 2.369)	0.969 (0.396, 2.370)
1–3 months	13.655 (0.381, 489.578)	0.554 (0.141, 2.177)	1.825 (0.298, 11.167)	1.652 (0.515, 5.301)
More than 3 months	0.267 (0.000, 564.092)	1.853 (0.259, 13.236)	8.834 (1.081, 72.180) *	0.113 (0.002, 7.731)
Use mosquito-repellent incense (ref: yes)	5.609 (0.276, 114.057)	0.783 (0.354, 1.733)	0.662 (0.223, 1.970)	0.865 (0.354, 2.114)
Use air fresheners (ref: yes)	0.913 (0.099, 8.395)	0.868 (0.428, 1.763)	1.435 (0.547, 3.768)	0.579 (0.270, 1.239)
Residential air pollution (ref: yes)	0.195 (0.018, 2.110)	0.439 (0.223, 0.864) *	0.521 (0.201, 1.351)	0.687 (0.329, 1.438)
Distance from home to road (ref: <0.5 km)	1.321 (0.137, 12.699)	0.988 (0.502, 1.944)	2.348 (0.926, 5.956)	1.040 (0.506, 2.138)
Closed to waste incineration plant, etc. (ref: yes)	0.001 (0.000, 0.175) **	0.871 (0.267, 2.842)	0.611 (0.128, 2.922)	0.928 (0.269, 3.202)

** *p* < 0.01, * *p <* 0.05. Bold numbers were indicated as statistically significant. ^1^ Adjusted for gender, age, height, premature birth, parental education level, parental occupation, father smoking, mother smoking, sleeping in own room, sleeping in own bed, household coal use, ventilation use when cooking, breast feeding, window opening and air conditioning use in different seasons, home renovation, presence of pets, presence of mold, mosquito coin use, air freshener use, air purifier use, kitchen type, parental bronchitis, intake of fruit, dairy, seafood and high-calorie food, doing physical exercise, ETS exposure and air exposure during commuting. ^2^ Children under 3 years old were excluded during the analyses. The association between humidity in different seasons and respiratory diseases is not shown in the table, since no valid data were obtained.

**Table 4:** Odds ratios and 95% confidence interval (95% CI) of annual heating time * opening window duration in different seasons for childhood respiratory disease, based on multivariate logistic regression models correlating all the confounders studied.

	Doctor-Diagnosed Asthma	Doctor-Diagnosed Bronchitis	Doctor-Diagnosed Pneumonia	Current Bronchitis
M0 + Annual heating time	3.362 (1.215, 9.298) *	1.267 (1.002, 1.601) *	0.908 (0.652, 1.263)	1.201 (0.942, 1.531)
M0 + Annual heating time * Window opening time in spring	1.621 (1.044, 2.519) *	1.016 (0.973, 1.061)	1.042 (0.982, 1.105)	1.024 (0.979, 1.070)
M0 + Annual heating time * Window opening time in winter	1.175 (0.945, 1.461)	1.028 (0.957, 1.104)	1.131 (1.016, 1.260) *	0.981 (0.921, 1.046)

The unit of window opening time and air conditioning time were all hour per day. The unit of annual heating time was months per year. Bold numbers were indicated as statistically significant. * indicates statistically significant difference with a *p* value < 0.05. Model 0: adjusted by age, sex, weight, district, ETS exposure, sleep in own room, sleep in own bed, household coal use, ventilation use when cooking, kitchen type, building type, paternal occupation, parental education level, breast feeding, parental asthma and bronchitis, doing physical exercise, residence air pollution, living close to waste incineration station, preterm birth, household mold, recently decoration, use incense, use air fresheners, have pets, residence within 0.5 km from the main road, air pollution exposure during commuting.

## Data Availability

Data with ID information removed to be available upon reasonable request made to the corresponding authors.

## Supplementary Materials

The following supporting information can be downloaded at: https://www.mdpi.com/article/10.3390/toxics11120964/s1, Table S1: Prevalence of childhood pneumonia with different potential influence factors.; Table S2: The stratification of respiratory diseases prevalence by various factors.
