# Supplementary material for: Asthma and Other Respiratory Diseases of Children in Relation to Personal Behavior, Household, Parental and Environmental Factors in West China
Source: Toxics. 2023 Nov 28;11(12):964. doi: 10.3390/toxics11120964 (PMC10747494; doi:10.3390/toxics11120964)
Supplement: Supplementary file 1 [file toxics-11-00964-s001.zip › toxics-2691324-supplementary.pdf]

Supplementary Materials: Asthma and Other Respiratory Diseases of Children in Relation to Personal Behavior, Household, Parental and Environmental Factors in West China

Changan Cao [1], Yuna Wang [2], Li Peng [3], Weiqi Wu [4], Huimin Yang [2] and Zhigang Li [5,*]

[1] School of Medicine, Xiamen University, Xiamen 361104, China; changan_cao@163.com
[2] School of Chemical and Environmental Sciences, YiLi Normal University, Yining 835000, China; yunawang99@163.com (Y.W.); yhm19991117@163.com (H.Y.)
[3] School of Energy and Environmental Engineering, University of Science of Technology Beijing, Beijing 10083, China; 42026184@xs.ustb.edu.cn
[4] Department of Geography, University College London, London WC1E 6BT, UK; zczqwwu@ucl.ac.uk
[5] Chinese Research Academy of Environmental Sciences, Beijing 100012, China
* Correspondence: lizg@craes.org.cn

Table S1. Prevalence of childhood pneumonia with different potential influence factors.

**Index**		Household coal use			Household mold		
		urban	rural	P	urban	rural	P
**Doctor-diagnosed Asthma**	**Yes**	2 (5.9%)	8 (18.6%)	*	6 (6.9%)	9 (9.9%)	
Doctor-diagnosed bronchitis	Yes	8 (23.5%)	10 (23.3%)		22 (25.3%)	21 (23.1%)	
Doctor-diagnosed pneumonia	Yes	5 (14.7%)	5 (11.6%)	*	11 (12.6%)	6 (6.6%)	*
Current bronchitis	Yes	6 (17.6%)	7 (16.3%)		21 (24.1%)	14 (15.4%)	*

* indicates statistically significant difference with a *P* value <0.05.

Table S2. The stratification of respiratory diseases prevalence by various factors.

	**Breastfeeding**	**Mother without asthma history**	**Mother ever got asthma**	**P**
Doctor-diagnosed Asthma	No	4 (11.4%)	1 (100%)	0.000
	Yes	16 (3.1%)	6 (60%)	
	**Breastfeeding**	**Mother without bronchitis history**	**Mother ever got bronchitis**	
Doctor-diagnosed Asthma	No	4 (12.9%)	1 (20%)	0.023
	Yes	18 (3.7%)	4 (9.5%)	
Doctor-diagnosed bronchitis	No	3 (9.7%)	1 (20%)	0.275
	Yes	76 (15.6%)	26 (61.9%)	
Doctor-diagnosed pneumonia	No	3 (9.7%)	0 (0)	1.000
	Yes	32 (6.6%)	11 (26.2%)	
Current bronchitis	No	2 (6.5%)	2 (25%)	0.805
	Yes	57 (11.7%)	19 (45.2%)	
